# Sense of Coherence in patients with stable schizophrenia: multidimensional determinants from a cross-sectional study of 438 inpatients in Guangzhou, China

**DOI:** 10.3389/fpsyt.2026.1750488

**Published:** 2026-06-15

**Authors:** Rongyu Liang, Zangrui Niu, Yuanyuan Huang, Xiezhen Chen, Fengchun Wu, Xiaodong Chen, Cuixia Liu, Xiaolei Shi, Gang Zeng

**Affiliations:** 1 Department of Geriatric Psychiatry, The Affiliated Brain Hospital, Guangzhou Medical University, Guangzhou, China; 2 Key Laboratory of Neurogenetics and Channelopathies of Guangdong Province and the Ministry of Education of China, Guangzhou Medical University, Guangzhou, China; 3 School of Nursing, Guangzhou Medical University, Guangzhou, China; 4 Department of Psychiatry, The Affiliated Brain Hospital, Guangzhou Medical University, Guangzhou, China; 5 Guangdong Engineering Technology Research Center for Translational Medicine of Mental Disorders, Guangzhou, China; 6 Chronic Psychiatry Department, The Affiliated Brain Hospital, Guangzhou Medical University, Guangzhou, China; 7 Cerebrovascular Disease Department, The Affiliated Brain Hospital, Guangzhou Medical University, Guangzhou, China; 8 Nursing Department, Guangzhou Chest Hospital Affiliated to Guangdong Pharmaceutical University, Guangzhou, China; 9 State Key Laboratory of Respiratory Disease, Guangzhou, China; 10 Institute of Tuberculosis, Guangzhou Medical University, Guangzhou, China

**Keywords:** Big Five Personality, inpatients, schizophrenia, Sense of Coherence, social support

## Abstract

**Introduction:**

Schizophrenia, a chronic psychiatric disorder, profoundly affects social functioning and quality of life. The Sense of Coherence (SOC), a core construct in salutogenic theory, plays a pivotal role in stress coping and recovery outcomes. While SOC has been studied in general populations, its determinants in stabilized schizophrenia inpatients remain underexplored. This study comprehensively investigated the Sense of Coherence (SOC) profile among stabilized schizophrenia inpatients by assessing its current status, exploring the interrelationships between SOC and demographic factors/Big Five personality traits (CBF-PI)/social support (SSRS), and identifying factors associated with SOC to inform potential personalized resilience-enhancing interventions. Findings may guide clinical strategies to enhance resilience and mental health recovery and potentially facilitate functional recovery in this population.

**Patients and methods:**

A cross-sectional study was conducted at a Guangzhou tertiary psychiatric hospital with 438 stabilized schizophrenia inpatients from November 2024 to February 2025. Data collection included demographic/clinical variables, Chinese Big Five Personality Inventory (CBF-PI), 13-item Sense of Coherence Scale (SOC-13), and the Social Support Rating Scale (SSRS). Statistical analyses were performed using SPSS version 27.0. Descriptive statistics were used to summarize the data and independent-samples t-tests and one-way analysis of variance (ANOVA) were employed to compare SOC scores across demographic and clinical groups. Pearson correlation analysis was conducted to examine the relationships between personality traits, social support, and sense of coherence and multiple linear regression analysis was utilized to identify independent factors influencing sense of coherence (*P* < 0.05).

**Result:**

The mean SOC score among the 438 stabilized schizophrenia inpatients was 53.35 ± 11.81. SOC correlated positively with all five dimensions of CBF-PI-B (P < 0.05), as well as subjective social support (P < 0.01), while showing inverse correlations with objective support (*p* < 0.01) and support utilization subscales (P < 0.01), Multiple regression identified personal income(yes), frequency of physical activity (≥3 times/week), and primary caregiver (Children) as significant SOC predictors.

**Conclusion:**

This study demonstrates that stabilized schizophrenia inpatients in a tertiary psychiatric setting exhibit generally lower Sense of Coherence (SOC) levels compared with healthy normative values and moderately lower than several outpatient schizophrenia samples. Higher SOC was significantly associated with having a personal income, frequent physical activity (≥3 times/week), having children as primary caregivers, and Chinese Big Five Personality traits. Notably, while subjective support was positively correlated with SOC, objective support and support utilization showed negative associations. Given the moderate internal consistency of the SSRS (Cronbach’s α = 0.626), the differential associations with social support dimensions should be interpreted cautiously. Concurrently, personality traits are relatively stable, their associations with SOC may inform indirect supportive strategies leveraging existing strengths rather than direct modification. These findings underscore the potential value of tailored interventions that enhance subjective support, promote structured recreational activities, and foster patients’ perceived coping resources. Further cross-sectional and longitudinal outpatient studies are warranted to validate these results and elucidate the mechanisms underlying the support paradox.

## 1 Introduction

Schizophrenia, a severe mental disorder, has become a major global public health concern. According to the World Health Organization (WHO) Global Burden of Disease (GBD) study, schizophrenia ranks among the top 15 causes of disability worldwide, affecting the quality of life and social functioning of more than 20 million individuals globally (1–3). Epidemiological data from China indicate a lifetime prevalence of approximately 0.6% (4), imposing a substantial burden on patients, families, and society. The clinical manifestations of schizophrenia are highly heterogeneous and complex, involving positive symptoms (e.g., hallucinations and delusions), negative symptoms (such as emotional blunting, diminished motivation, and social withdrawal), cognitive impairments (including deficits in attention, memory, and executive function), and emotional symptoms (like depression and anxiety) (5). Following acute - phase treatment, patients typically transition into a stable phase in which positive symptoms are effectively controlled. However, persistent negative symptoms and cognitive impairments continue to pose significant obstacles to social reintegration and recovery (6, 7). Consequently, facilitating psychosocial rehabilitation and functional recovery among patients in the stable phase constitutes a crucial challenge in the realm of mental healthcare.

## 2 Background

Against such a backdrop, the salutogenic model, which was proposed by sociologist Aaron Antonovsky, offers a highly valuable framework for mental health rehabilitation (8). This model poses a challenge to the traditional pathogenic perspective by centering on the factors that empower individuals to uphold their health amid stress. At the core of the salutogenic model lies the concept of Sense of Coherence (SOC), which is defined as an individual’s overall orientation that renders life comprehensible, manageable, and meaningful (9, 10). SOC consists of three interrelated dimensions: comprehensibility (the perception that both internal and external stimuli are structured and predictable), manageability (the belief in having the resources to meet demands), and meaningfulness (the sense that the challenges in life are worth the investment) (9, 11). Unlike self-efficacy, which primarily reflects confidence in performing specific behaviors or tasks) (42), or personal recovery, which focuses on subjective experiences of hope, empowerment, and life satisfaction as outcomes (43), SOC represents a more generalized, dispositional orientation toward life stressors. Instead of merely aiming to eradicate stress, SOC enables cognitive reframing of stressors as manageable challenges, functioning as a crucial internal resource for coping and fostering mental health (9, 12). In the context of stabilized schizophrenia, where persistent negative symptoms and cognitive deficits limit task-specific efficacy and full functional recovery (35, 44), SOC may offer unique explanatory power by capturing how patients maintain a sense of coherence despite enduring impairments, thereby providing a complementary perspective to existing recovery-oriented models (10, 13),.

Although previous studies have identified SOC as a significant positive predictor of mental health outcomes in individuals with schizophrenia (13), many investigations have been limited to univariable analyses, neglecting the complex interactive mechanisms underlying SOC. The Chinese Big Five Personality traits, representing stable psychological tendencies, and social support, a critical external resource, have been regarded as key factors associated with SOC (14, 15). Specifically, Neuroticism and Extraversion have been highlighted as stable personality dimensions affecting SOC (16). Neuroticism, characterized by negative emotionality, may impair one’s sense of comprehensibility and manageability (16–18), whereas Extraversion, through active social engagement, has been linked to higher manageability and meaningfulness. Similarly, social support encompasses subjective (perceived care), objective (tangible aid), and utilized support; subjective support has been particularly associated with higher meaningfulness, while objective support has shown associations with manageability (19). It is likely that complex synergistic and regulatory interactions exist among Chinese Big Five Personality traits, social support, and SOC, rather than a simple additive effect.

Despite the presence of these theoretical foundations, significant research gaps still persist (1): Limited investigation of SOC among hospitalized patients with stabilized schizophrenia (20); (2) Insufficient exploration of interactive mechanisms among key variables; and (3) inadequate analysis of distinct patterns of association of various social support dimensions on SOC components. Accordingly, this study aimed to (1) evaluate SOC levels and their multidimensional correlates (Chinese Big Five Personality traits and social support dimensions) among hospitalized patients with stabilized schizophrenia; and (2) identify independent factors associated with SOC using multivariable modeling. The findings are expected to inform targeted interventions to strengthen SOC and thereby support mental health recovery and functional outcomes in stabilized schizophrenia patients during hospitalization and the rehabilitation process.

## 3 Materials and methods

### 3.1 Subjects

This cross-sectional study employed convenience sampling to recruit 438 patients with stable schizophrenia who were hospitalized at a tertiary-level mental health hospital in Southern China between November 2024 and February 2025.

Inclusion criteria were as follows: (1) diagnosis of schizophrenia according to the International Classification of Diseases, 10th Revision (ICD-10); (2) age ≥18 years; (3) inpatient status in the stable phase, inpatient status in the stable phase, receiving systematic pharmacological treatment, and a Positive and Negative Syndrome Scale (PANSS) score below 60. This cutoff is widely adopted in previous research to indicate mild or no symptomatic severity and suitability for psychosocial assessments, based on established PANSS severity thresholds (21, 46); (4) ability to communicate in Mandarin or Cantonese and to comprehend and complete the questionnaires accurately; and (5) voluntary participation with signed informed consent.

Exclusion criteria included: (1) intellectual disability; (2) concomitant organic brain disorders such as epilepsy or dementia; and (3) withdrawal from the study during the data collection period. The study protocol was approved by the hospital’s ethics committee, and all participants provided written informed consent prior to participation.

To ensure the accuracy and reliability of the results, the sample size generally should be 5–10 times the number of independent variables (41). Specifically, the sample size calculation formula could be expressed as: sample size = number of independent variables × (5-10) (41). This study used 19 variables. Therefore, the preliminary estimate required 190 cases. Considering a 15% rate of invalid data, the final estimate needed 219 samples. This study included 438 subjects. All subjects met the inclusion and exclusion criteria.

### 3.2 Questionnaire

#### 3.2.1 General information questionnaire

Demographic and clinical data were collected using a general information questionnaire, which included age, gender, residence, type of medical insurance, educational attainment, marital status, number of children, sleep quality, smoking status, employment status prior to admission, personal income source and level, monthly household income, presence of a caregiver and primary caregiver, duration since illness onset, previous hospitalizations, frequency of exercise, and frequency of recreational activities.

#### 3.2.2 Sense of Coherence Scale

The Sense of Coherence Scale (SOC-13), developed by Antonovsky (9)and adapted into Chinese by Leiping Bao et al. (22), was used to assess individuals’ mental health. The SOC scale has demonstrated acceptable construct and predictive validity in patients with schizophrenia (8, 13), justifying its application in stabilized inpatients in the present study. The scale comprises three dimensions: comprehensibility, manageability, and meaningfulness (22). It contains 13 items scored on a 7-point Likert scale, including 5 reverse-scored items (22). Total scores range from 13 to 91, with higher scores indicating stronger SOC (22). In this study, the scale demonstrated good internal consistency with a Cronbach’s α of 0.876.

#### 3.2.3 Chinese Big Five Personality Inventory

Personality traits were measured using the Chinese Big Five Personality Inventory-B (CBF-PI-B), revised by Mengcheng Wang et al. (23). The questionnaire assesses five personality dimensions: conscientiousness, agreeableness, neuroticism, extraversion, and openness. It contains 40 items (33 positively keyed, 7 negatively keyed) rated on a 6-point Likert scale from 1 (completely disagree) to 6 (completely agree) (23). Scores for each dimension range from 8 to 48, with higher scores indicating stronger personality traits (23). The Cronbach’s α for this scale in this study was 0.887.

#### 3.2.4 Social Support Rating Scale

Social support was evaluated using the Social Support Rating Scale (SSRS), developed by Shuiyuan Xiao (24). The scale includes three dimensions: objective support, subjective support, and support utilization, comprising 10 items in total (24). Total scores range from 12 to 66, with higher scores indicating greater social support (24). The Cronbach’s α coefficient in this study was 0.626.

### 3.3 Data collection

All questionnaires were administered, filled out, and gathered on-site. Before distribution, the researchers elucidated the study’s objectives and procedures to the patients, who subsequently provided written informed consent. Participants were given instructions in their preferred language (Mandarin or Cantonese) and received on-site assistance throughout the entire process. All questionnaires are self-reported by participants. Any queries raised by the participants were responded to uniformly by the researchers. The completed questionnaires were promptly reviewed for completeness. A total of 470 questionnaires were distributed, of which 446 were completed, yielding a completion rate of 94.89%. Analysis of the 8 incomplete questionnaires showed that three patients withdrew after completing two-thirds of the questionnaire and the remaining 5 incomplete questionnaires were discarded due to more than half of incomplete responses, resulting in a final dataset comprising 438 responses.

### 3.4 Statistical analysis

Data entry and statistical analyses were carried out utilizing SPSS version 27.0. Categorical variables were summarized in the form of frequencies and percentages. Meanwhile, continuous variables that adhered to a normal distribution were presented as means ± standard deviations ( $\bar{X}\pm S$).

Group comparisons were made through independent-samples *t*-tests and one-way analysis of variance (ANOVA). Pearson correlation analysis was performed to examine the bivariate relationships between SOC total score and the domains/subscales of CBF-PI and SSRS, as well as among the psychosocial variables themselves, in order to identify relevant associations and inform subsequent multivariable modeling. Multiple linear regression analysis was employed to identify the factors associated with sense of coherence. To account for potential confounding by residual symptom domains, the five-factor structure of the PANSS (positive, negative, disorganized thought/conceptual disorganization, excitement, and depression/anxiety) was calculated based on Wallwork et al. and included as covariates in the multiple linear regression model (45, 46). *P* < 0.05 was considered statistically significant.

## 4 Ethics approval and informed consent

This study obtained ethical approval from the IRB of the Affiliated Brain Hospital of Guangzhou Medical University (approval number: 2020028). Written informed consent was obtained from all participants before inclusion. The entire study was carried out in accordance with the Declaration of Helsinki.

## 5 Results

### 5.1 General information of the respondents

A total of 438 patients were enrolled in the study. **Table 1** presents the demographic and clinical characteristics of the 438 participants, along with univariable comparisons of SOC scores across groups using independent-samples *t-*tests and one-way ANOVA. Statistically significant differences (*P* < 0.05) were observed for several factors, detailed values are reported in **Table 1**.

**Table 1:** Univariable analysis of sense of coherence in patients with stable-phase schizophrenia.

Characteristic	Frequency (%)	SOC score ( $\bar{X}\pm S$)	F	P
Age (years)			6.581	< 0.001**
18–30	50 (11.4)	49.18 ± 14.37		
30–50	164 (37.4)	51.48 ± 12.03		
50–70	161 (36.8)	55.40 ± 11.16		
>70	63 (14.4)	53.35 ± 11.81		
Gender			0.391	0.532
Male	220 (50.2)	52.99 ± 11.71		
Female	218 (49.8)	53.70 ± 11.94		
Residence			1.763	0.173
City	186 (42.5)	54.32 ± 11.44		
County	161 (36.8)	53.28 ± 11.94		
Rural	91 (20.8)	51.49 ± 12.24		
Medical Insurance Type			0.518	0.763
Self-Paid	54 (12.3)	53.72 ± 11.48		
Rural Medical Insurance	102 (23.3)	53.35 ± 12.78		
Employee Medical Insurance	232 (53.0)	53.08 ± 11.83		
Publicly Funded Medical Insurance	23 (5.3)	51.65 ± 10.43		
Lifetime Hospitalization***	3 (0.7)	59.67 ± 3.79		
Lifetime Hospitalization (Insurance)	24 (5.5)	55.35 ± 10.25		
Educational Level			0.396	0.812
Primary school or below	41 (9.4)	53.56 ± 10.57		
Junior high school	148 (33.8)	53.53 ± 11.16		
Senior high school/Technical secondary school	157 (35.8)	52.76 ± 11.52		
College	58 (13.2)	54.86 ± 13.38		
University or above	34 (7.8)	52.44 ± 14.66		
Marital Status			5.054	0.002**
Single	255 (58.2)	51.66 ± 10.86		
Married	104 (23.7)	56.85 ± 12.65		
Divorced	71 (16.2)	54.28 ± 12.80		
Widowed	8 (1.8)	53.25 ± 12.00		
Number of Children			4.382	0.005**
None	294 (67.1)	52.08 ± 11.08		
1	107 (24.4)	56.21 ± 12.55		
2	22 (5.0)	57.59 ± 14.25		
≥3	15 (3.4)	51.40 ± 12.83		
Sleep Quality			0.743	0.563
Excellent	85 (19.4)	57.46 ± 11.72		
Good	189 (43.2)	50.94 ± 11.53		
Average	115 (26.3)	53.69 ± 11.72		
Poor	33 (7.5)	54.45 ± 11.65		
Very poor	16 (3.7)	55.19 ± 11.01		
Smoking Status			0.627	0.429
Never	288 (65.8)	53.02 ± 11.43		
Smoking	150 (34.2)	53.97 ± 12.54		
Employment Status Before Admission			6.615	<0.001**
Employed	74 (16.9)	53.04 ± 12.16		
Self-employed	40 (9.1)	46.33 ± 11.72		
Unemployed	195 (44.5)	52.69 ± 11.60		
Retired	117 (26.7)	56.77 ± 10.94		
Other	12 (2.7)	55.92 ± 10.93		
Has Personal Income Source			11.327	<0.001**
Yes	206 (47.0)	55.34 ± 12.45		
No	232 (53.0)	51.58 ± 10.95		
Personal Monthly Income (yuan)			0.656	0.657
<2000	50 (11.4)	53.34 ± 11.12		
2001–3000	49 (11.2)	54.96 ± 11.26		
3001–5000	63 (14.4)	55.71 ± 13.64		
5001–8000	28 (6.4)	58.04 ± 13.63		
8001–10000	6 (1.4)	59.00 ± 9.47		
>10000	8 (1.8)	53.88 ± 17.07		
Missing value	234(53.4)	—		
Monthly Household Income (yuan)			1.628	0.184
<5000	46 (10.5)	53.78 ± 12.28		
5001–10000	103 (23.5)	56.32 ± 13.24		
10001–15000	35 (8.0)	51.95 ± 12.62		
>15000	12 (2.7)	58.06 ± 16.90		
Missing value	242(55.3)	—		
Accompanied by Caregiver			0.400	0.527
Yes	44 (10.1)	54.41 ± 14.95		
No	393 (89.7)	53.22 ± 11.44		
Missing value	1(0.2)	—		
Primary Caregiver			6.062	<0.001**
Parents	38 (8.7)	53.68 ± 11.93		
Children	50 (11.4)	61.6 ± 11.13		
Spouse	22 (5.0)	51.87 ± 13.52		
Siblings	24 (5.5)	50.54 ± 11.29		
Friends	3 (0.7)	55.33 ± 6.81		
Other	300 (68.5)	52.3 ± 11.79		
Missing value	1(0.2)	—		
Duration of Mental Illness (years)			4.157	0.003**
≤10	80 (18.3)	57.31 ± 14.10		
11–20	90 (20.5)	50.87 ± 11.98		
21–30	96 (21.9)	51.39 ± 11.01		
31–40	112 (25.6)	54.16 ± 10.58		
>40	60 (13.7)	53.40 ± 10.35		
Number of Previous Hospitalizations			1.366	0.256
0–5	161 (36.8)	54.31 ± 13.17		
6–10	162 (37.0)	52.18 ± 10.95		
≥11	115 (26.3)	53.64 ± 10.91		
Frequency of Physical Activity			1.142	0.332
None	192 (43.8)	53.18 ± 11.98		
1–2 times/week	140 (32.0)	53.77 ± 11.72		
3–4 times/week	53 (12.1)	55.13 ± 12.53		
≥5 times/week	53 (12.1)	51.06 ± 10.61		
Frequency of Recreational Activities			5.553	0.004**
Never	146 (33.3)	51.02 ± 12.03		
Occasionally (<3 times/week)	242 (55.3)	54.02 ± 11.42		
Frequently (≥3 times/week)	50 (11.4)	56.86 ± 11.98		

**P<0.01.

***Lifetime hospitalization pertains to an insurance product or medical service commitment that offers financial protection for the costs of hospitalization treatment arising from illness or accidental injury over the entire lifespan of the insured individual.

### 5.2 Scores of Senses of Coherence Scale, Chinese Big Five Personality Inventory and Social Support Rating Scale in patients with stable-phase schizophrenia

The mean Sense of Coherence (SOC-13) score among the 438 hospitalized patients with stable-phase schizophrenia was 53.35 ± 11.81. The mean total score for the Chinese Big Five Personality Inventory (CBF-PI) was 133.23 ± 31.62, and the Social Support Rating Scale (SSRS) score was 27.20 ± 7.44. Detailed scores for each dimension are presented in **Table 2**.

**Table 2:** Pearson correlation coefficients between SOC-13, CBF-PI-B and SSRS in patients with stable-phase schizophrenia. *Reverse-scored Neuroticism, **All *P*<0.001.

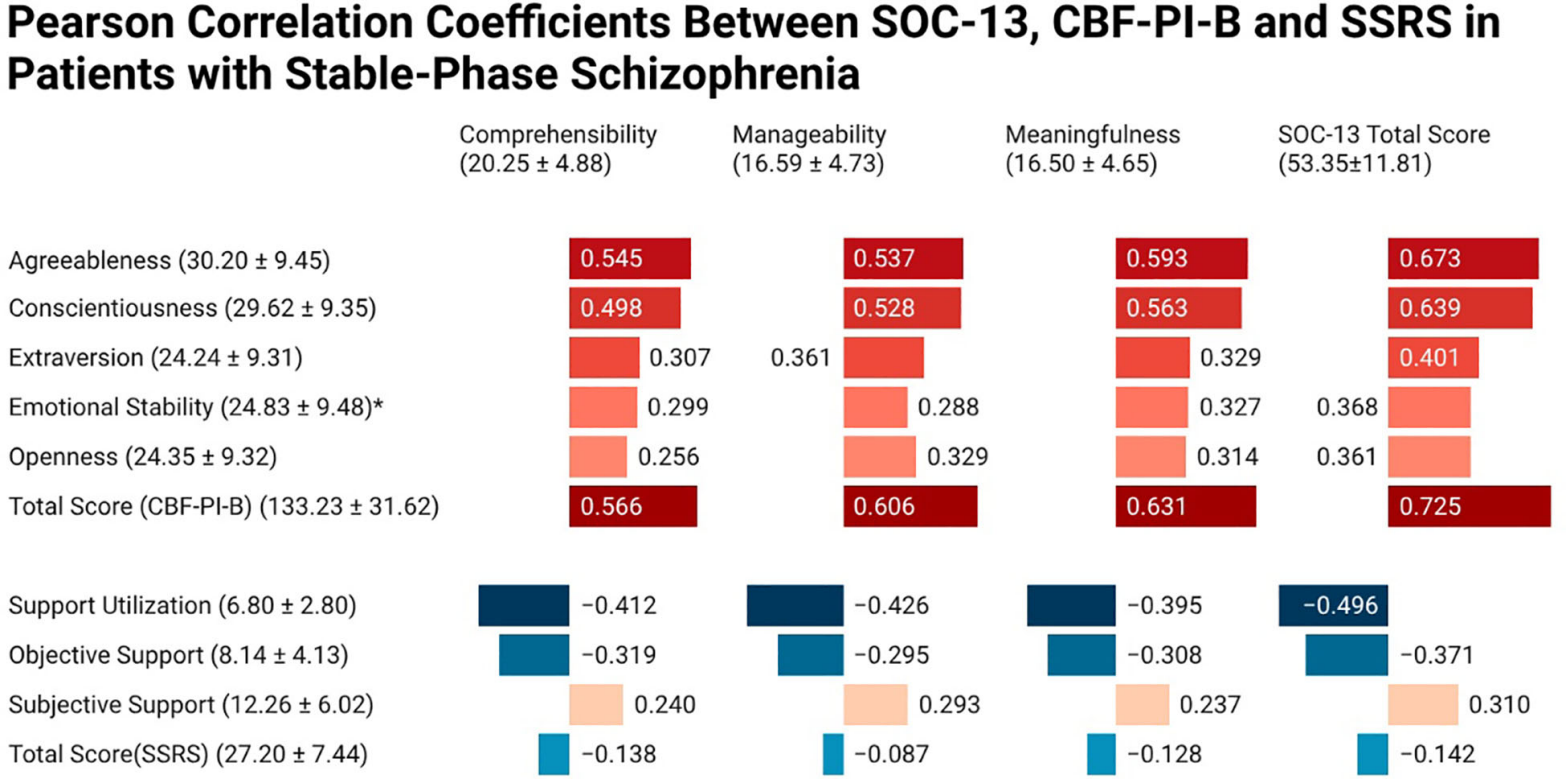

### 5.3 Univariable analysis of Sense of Coherence in patients with stable-phase schizophrenia

The results showed that total Sense of Coherence (SOC) scores differed significantly according to age, marital status, parental status, employment status prior to hospitalization, primary caregiver, personal income status, duration of illness, and frequency of recreational activities (all *p* < 0.05), as detailed in **Table 1**.

### 5.4 Correlation analysis between Sense of Coherence, Chinese Big Five Personality traits, and social support in patients with stable-phase schizophrenia

The Pearson correlation analysis demonstrated that the total SOC score and its subdimensions exhibited positive correlations with scores on the Chinese Big Five Personality Inventory (CBF - PI) and the subjective support dimension of the Social Support Rating Scale (SSRS). In contrast, negative correlations were identified between SOC and objective support, support utilization, and total social support scores. These findings are concisely summarized in **Table 2**.

### 5.5 Multiple linear regression analysis of factors influencing Sense of Coherence in patients with stable schizophrenia

A multiple linear regression analysis was conducted, with the total SOC score serving as the dependent variable. A multiple linear regression analysis was conducted, with the SOC score serving as the dependent variable. The independent variables encompassed those that exhibited statistical significance in univariable and Pearson correlation analyses. Categorical variables were entered as dummy variables with clinically and statistically justified reference categories. Specifically, for marital status, “divorced” and “widowed” were merged into a single category “divorced/widowed” due to small sample sizes and similar psychosocial implications; for number of children, “2 children” and “≥3 children” were merged into “≥2 children” to address low frequency and comparable functional impact in this population. Other low-frequency categories were similarly merged where appropriate with clear justification to avoid sparse data issues and enhance model stability. The comprehensive results of the multiple linear regression analysis are presented in **Table 3**. The comprehensive results of the multiple linear regression analysis are presented in **Table 3**.

**Table 3:** Multiple linear regression analysis of factors influencing sense of coherence in patients with stable schizophrenia.

Variable		Unstandardized coefficient		Standardized coefficient	*t*	*P*
		*B*	*SE*	*β*		
Constant		28.664	2.821	—	10.161	<0.001**
Age		0.008	0.027	0.011	0.316	0.752
Marital Status	Single (Reference)	—	—	—	—	—
	Married	1.306	1.243	0.047	1.051	0.294
	Divorced/Widowed	2.071	1.111	0.068	1.864	0.063
Number of Children	None (Reference)					
	1	-0.384	1.114	-0.014	-0.345	0.730
	≥2	0.159	1.435	0.004	0.111	0.912
Employment Status Before Admission	Employed (Reference)	—	—	—	—	—
	Self-employed	-2.62	1.369	-0.064	-1.914	0.056
	Unemployed	-0.188	0.993	-0.008	-0.189	0.850
	Retired	0.504	1.102	0.019	0.457	0.648
	Other	-0.665	2.179	-0.009	-0.305	0.760
Has Personal Income Source	No (Reference)	—	—	—	—	—
	Yes	1.454	0.714	0.062	2.036	0.042*
Primary Caregiver	Other (Reference)	—	—	—	—	—
	Parents	0.252	1.279	0.006	0.197	0.844
	Children	2.196	1.112	0.059	1.974	0.049*
	Spouse	-1.372	1.765	-0.025	-0.777	0.438
	Siblings	-0.293	1.479	-0.006	-0.198	0.843
	Friends	-3.323	4.053	-0.023	-0.82	0.513
Duration of Mental Illness (years)		-0.057	0.034	-0.067	-1.692	0.091
Frequency of Physical Activity	None (Reference)	—	—	—	—	—
	<3 times/week	0.246	0.734	0.01	0.336	0.737
	≥3 times/week	3.521	1.17	0.094	3.009	0.003**
CBF-PI-B	Agreeableness	0.299	0.049	0.24	6.13	<0.001**
	Conscientiousness	0.334	0.047	0.264	7.05	<0.001**
	Extraversion	0.122	0.04	0.096	3.053	0.02*
	Emotional Stability***	0.117	0.038	0.094	3.073	0.02*
	Openness	0.133	0.039	0.105	3.433	<0.001**
SSRS	Support Utilization	-0.819	0.133	-0.194	-6.166	<0.001**
	Objective Support	-0.366	0.087	-0.128	-4.211	<0.001**
	Subjective Support	0.246	0.058	0.126	4.276	<0.001**

*R²* = 0.685, adjusted *R²* = 0.666, *F* = 35.795; *P < 0.05, **P < 0.01, ***Reverse-scored Neuroticism.

## 6 Discussions

### 6.1 Overall low level of Sense of Coherence in stabilized schizophrenia inpatients

This study found that the mean total Sense of Coherence (SOC) score among 438 stabilized schizophrenia inpatients was 53.35 ± 11.81, indicating a relatively low level (SOC-13 scale low range: 13–63) (22). Compared with normative data from healthy Chinese adults (SOC total score mean ≈ 65–68) (47, 48), this value is substantially lower. When benchmarked against Linyin Jiang et al.’s study on patients in the rehabilitation phase of schizophrenia, our inpatient mean is similar to or slightly higher than that reported in recovery-phase outpatients (SOC total score 52.21 ± 11.35) (26), but markedly lower than community-dwelling stable patients in some European cohorts (SOC total score ≈58–62) (13). Eriksson et al. reported that the mean SOC-13 score in patients with major depressive disorder (MDD) during the stable or remission phase was approximately 61.2 ± 12.4. These comparisons suggest that the particularly low SOC observed in stabilized schizophrenia inpatient sample may reflect the more pervasive and enduring impact of schizophrenia on comprehensibility, manageability, and meaningfulness. Even in clinically stable phases, schizophrenia involves chronic cognitive deficits and social withdrawal, which fundamentally undermine patients’ sense of meaning (27), This low SOC level may be attributed to multidimensional impairments in perception, cognition, and emotion intrinsic to schizophrenia, which weaken patients’ capacity to integrate internal and external information, thereby diminishing comprehensibility and manageability (21, 25). Therefore, interventions should go beyond symptom management and incorporate psychosocial strategies that can enhance patients’ understanding of their condition and cultivate their sense of control.

### 6.2 Analysis of key factors influencing Sense of Coherence

Multiple linear regression analysis identified personal income(yes), frequency of physical activity (≥3 times/week), and primary caregiver (Children) as significant predictors of SOC. Patients having a personal source of income demonstrated higher SOC levels. Economic stability acts as a crucial resistance resource, enhancing the “manageability” component by providing patients with the means to solve daily problems and reducing perceived life burden (31). Physical activity was another strong predictor; patients who exercised three or more times a week had significantly higher SOC. Regular physical activity not only improves physical health but also serves as a behavioral activation strategy that alleviates negative symptoms and fosters a sense of meaningfulness and achievement (28). Regarding family structure, patients whose primary caregivers were their children showed significantly higher SOC compared to those cared for by others. This may suggest that support from the younger generation provides a stronger sense of family continuity and emotional reliance, or it may imply a specific family dynamic where the patient retains a respected parental role, thereby bolstering their self-worth and meaningfulness (28). These findings suggest that personalized interventions should focus on vocational rehabilitation to improve economic independence and the integration of structured physical exercise programs into routine care.

### 6.3 Influence of Chinese Big Five Personality on Sense of Coherence

Multiple linear regression analysis demonstrated that all five dimensions of CBF-PI-B were significant independent predictors of SOC in stabilized schizophrenia inpatients. Among these, Conscientiousness and Agreeableness exhibited the strongest positive associations. Patients with high Conscientiousness typically possess strong organizational skills, self-discipline, and goal-directed behaviors. In the context of long-term hospitalization, these traits likely facilitate better adherence to treatment regimens and daily routines, thereby directly enhancing the “Manageability” component of SOC (29, 30). Similarly, Agreeableness, characterized by altruism, cooperation, and trust, is crucial for social adaptation in a ward environment. Patients with higher agreeableness are more likely to establish positive relationships with healthcare providers and fellow patients (31). These supportive interpersonal bonds foster a sense of belonging and emotional security, which strengthens the “Meaningfulness” of their recovery experience (31, 32). Openness and Extraversion also served as positive predictors. Openness allows patients to better adapt to the changes and cognitive challenges posed by the illness, enhancing “Comprehensibility” (27), while Extraversion drives patients to actively engage in social interactions and rehabilitation activities (27). Notably, the dimension of Emotional Stability (reverse-scored Neuroticism) showed a significant positive association with SOC. This finding is consistent with the theoretical core of SOC, where emotional calmness and reduced anxiety allow patients to better perceive their environment as comprehensible and manageable (33, 34). While the strong associations between SOC and certain Chinese Big Five Personality (particularly conscientiousness and agreeableness) are noteworthy, personality traits are generally considered to be relatively stable dispositions in adulthood with limited short-term malleability (29, 32). The primary focus of intervention should remain on modifiable factors (such as subjective social support and recreational activity frequency) rather than attempting to alter stable personality characteristics. Concurrently, these associations may serve as prognostic indicators or supportive strategies (35), including leveraging patients’ existing strengths in conscientiousness (e.g., goal-directedness and reliability) to enhance treatment adherence and self-management skills, or using agreeableness-related prosocial tendencies to facilitate engagement in peer support or group-based therapies.

### 6.4 Dual effects of social support on Sense of Coherence

This study revealed that subjective social support was positively correlated with SOC, consistent with the view that perceived emotional care may contribute to mental resilience (36). In contrast, support utilization demonstrated a significant negative correlation with SOC (Cronbach’s α = 0.626). This negative association suggests that higher utilization of support does not necessarily equate to better psychological coherence, one possible interpretation is that patients with lower SOC may rely more on external assistance, potentially reflecting perceived limitations in personal coping resources, particularly in the manageability domain (38). Patients with lower SOC, particularly those scoring low on the “manageability” component, may perceive their internal resources as insufficient to handle daily stressors. Consequently, they feel compelled to frequently seek and utilize assistance from others as a compensatory strategy (38). In this context, high support utilization serves as a marker of psychological dependency and a lack of autonomy, rather than a proactive mobilization of resources (39). Conversely, patients with higher SOC tend to possess stronger self-efficacy and problem-solving abilities, allowing them to manage challenges independently without constantly relying on external aid. Therefore, clinical interventions should not merely aim to increase the quantity of support provided but should prioritize the quality of support and patient empowerment. Strategies should guide caregivers to provide “autonomy-supportive” help—assisting patients in building their own coping skills—rather than fostering reliance on continuous external intervention (37, 40).

### 6.5 Limitations and future directions

This study is subject to several limitations. First, the cross-sectional design precludes the determination of causal relationships between SOC and the identified influencing factors. Second, the use of convenience sampling from a single psychiatric center may introduce selection bias, potentially limiting the generalizability of the findings to patients in different geographic regions or distinct healthcare settings. Third, although we examined multiple factors, the study did not incorporate certain potentially pertinent variables, such as objective measures of cognitive function, detailed family functioning assessments, and medication side effects. Fourth, functional outcomes (e.g., social, occupational, and independent living functioning) were not directly assessed in this study. Any inference regarding direct impact on functional recovery should therefore be considered tentative and requires validation through studies that include objective functional outcome measures. Fifth, while we defined clinical stability using a PANSS total score of<60—a widely adopted threshold for mild symptom severity—this approach may oversimplify the heterogeneous nature of schizophrenia symptoms. As highlighted by recent literature (45, 49), collapsing distinct symptom domains into a single total score risks obscuring the specific impact of negative symptoms (e.g., avolition, anhedonia, blunted affect) on psychological constructs such as SOC. It is plausible that the core components of SOC—particularly “manageability” and “comprehensibility”—could be directly confounded by residual negative symptoms, rather than reflecting purely independent psychological or salutogenic factors. Consequently, we cannot entirely rule out that some of the variance in SOC scores may be attributable to unmeasured negative or depressive symptoms. Sixth, the Cronbach’s alpha coefficient for the SSRS in the present sample was 0.626, which is below the conventional threshold of 0.70 commonly recommended for research instruments, and may have reduced the precision of the social support measurement.

Despite these limitations, the study provides several meaningful contributions. The study represents one of the larger inpatient-based investigations of SOC in stabilized schizophrenia in China (n=438). It simultaneously examined relatively stable personality traits (via the Chinese Big Five Personality Inventory) and the multidimensional structure of social support (objective support, subjective support, and support utilization), and it revealed an intriguing “support paradox” — subjective support positively associated with SOC while objective support and support utilization showed unexpected negative associations — which merits further investigation.

To advance beyond the current findings, future research could first apply more advanced cross-sectional techniques, such as mediation analysis, moderation analysis, or structural equation modeling, to clarify potential pathways, interactive effects, and confounding influences (particularly the role of specific symptom dimensions like negative symptoms) among personality, social support, recreational activity frequency, and SOC using the existing data. Subsequently, prospective longitudinal studies — ideally conducted in outpatient or community settings — are strongly recommended to track changes in SOC over time, establish temporal relationships, and elucidate underlying mechanisms (including the counterintuitive effects of objective support and support utilization). Such designs would provide stronger evidence to guide the development of targeted, recovery-oriented interventions for this population.

## 7 Conclusion

Sense of Coherence (SOC) levels among stabilized schizophrenia inpatients are generally low and are primarily correlated with by personal income(yes), frequency of physical activity (≥3 times/week), primary caregiver (Children), Chinese Big Five personality traits and Social Support. SOC showed positive correlations with all five personality dimensions—with conscientiousness and agreeableness showing particularly strong links—as well as subjective social support. Conversely, objective social support and support utilization exhibited negative correlations with SOC, suggesting that excessive reliance on external help may reflect a lack of internal coping resources. Therefore, clinical interventions should move beyond symptom management to adopt personalized, salutogenic strategies. Priorities should include (1): implementing vocational rehabilitation to enhance economic independence; (2) integrating structured physical exercise programs into routine care; (3) strengthening family-based emotional support; and (4) fostering patient autonomy to transform passive dependency into active coping. Future research should address the limitations of convenience sampling and cross-sectional design by employing multi-center longitudinal studies and advanced analytical methods to further elucidate the causal mechanisms underlying SOC dynamics.

## Data Availability

The raw data supporting the conclusions of this article will be made available by the authors, without undue reservation.
